# Colorectal Cancer: The Contribution of CXCL12 and Its Receptors CXCR4 and CXCR7

**DOI:** 10.3390/cancers14071810

**Published:** 2022-04-02

**Authors:** Aïssata Aimée Goïta, Dominique Guenot

**Affiliations:** INSERM U1113/Unistra, IRFAC—Interface de Recherche Fondamentale et Appliquée en Cancérologie, 67200 Strasbourg, France; goitaaissata@yahoo.fr

**Keywords:** colorectal cancer, chemokine, ACKR3, metastasis, microenvironment, signaling pathways, epigenetics, prognosis, therapy, resistance

## Abstract

**Simple Summary:**

Many signaling pathways are involved in cancer progression, and among these pathways, the CXCL12 axis and its two receptors CXCR4 and CXCR7 are well described for many cancers. This review presents the current knowledge on the role played by each of the actors of this axis in colorectal cancer and on its consideration in the development of new therapeutic strategies.

**Abstract:**

Colorectal cancer is one of the most common cancers, and diagnosis at late metastatic stages is the main cause of death related to this cancer. This progression to metastasis is complex and involves different molecules such as the chemokine CXCL12 and its two receptors CXCR4 and CXCR7. The high expression of receptors in CRC is often associated with a poor prognosis and aggressiveness of the tumor. The interaction of CXCL12 and its receptors activates signaling pathways that induce chemotaxis, proliferation, migration, and cell invasion. To this end, receptor inhibitors were developed, and their use in preclinical and clinical studies is ongoing. This review provides an overview of studies involving CXCR4 and CXCR7 in CRC with an update on their targeting in anti-cancer therapies.

## 1. Cancer Colorectal

### 1.1. Epidemiology

Colorectal cancer (CRC) is the third most common cancer in the world, with an annual estimate in 2020 of 1,148,515 new cases affecting both men and women. Because most patients are diagnosed at metastatic stages of the disease [1], it is the cause of 576,858 deaths per year, making it the second most deadly cancer. Similar to many cancers, the etiology of CRC involves a variety of environmental and individual risk factors, including genetic causes, chronic disease, lifestyle, and age [2].

An average risk is attributed to men and women over 50 years of age with no known predisposing factors. In absence of genetic factors or family history, environmental factors such as diet, a sedentary lifestyle, alcohol, and tobacco abuse influence the development of CRC [3,4]. The high risk, about 20% of the general population, considers family (familial adenomatous polyposis or FAP or Lynch syndrome) and personal history. Thus, this risk is two to five times higher than the average risk of developing CRC for people who have had an adenoma >1 cm, or with at least one first-degree relative who has developed colorectal adenomas or CRC [5]. The risk is also elevated for people affected by a chronic inflammatory bowel disease (IBD) such as ulcerative colitis or Crohn’s disease [5].

### 1.2. Molecular Definition of Colorectal Cancer

CRC occurs and progresses because of an accumulation of sequential mutations and/or genomic abnormalities. Molecular biology techniques have classified CRCs into three major phenotypes according to the abnormalities identified [6]. Tumors with a Chromosome INstability phenotype (CIN) or a MicroSatellite Stability phenotype (MSS) are the most frequently observed (80–85%) [7]. This instability is a result of loss or gain of chromosomes or chromosome fragments leading to loss of tumor suppressor genes or gain of oncogenes [8]. Examples include the loss of chromosomes 5, 17 and 18 on which the APC (5q21), TP53 (17p13) and SMAD2-3 (18q21) genes are located, respectively, and the gains on chromosome 8 for the C-MYC gene on 8q24 [8,9,10,11,12].

The second phenotype represents tumors characterized by MicroSatellite Instability (MSI) and is present in 15–20% of CRCs [13]. This phenotype is characterized by a deficiency in the base MisMatch Repair (MMR) system during replication [14]. This defect results in an accumulation of mutations in microsatellites and repeated sequences of one to twenty nucleotides in the coding region of certain genes involved in colorectal carcinogenesis [15]. Among the genes affected are mainly MLH1 and MSH2, which are also associated with Lynch syndrome [16,17]. The MSI phenotype is also classified into MSI-High and MSI-Low.

A third phenotype has been established by observing methylation/hypermethylation of CG repeat sequences or CpG (cytosine–phosphate–guanine) islands in the promoter regions of some genes, thus repressing their transcriptional expression [18]. These repressions typically affect many tumor suppressor genes such as MLH1, CDKN2A [19]. The latter phenotype can be found associated with either of the previous two phenotypes, as 12% of CIMP cases are found associated with the MSI phenotype and 8% in MSS phenotypes [20].

In general, CRC survival depends directly on the stages. Thus, the overall survival at 5 years for all stages combined is 63%, and the chance of cure is almost total for stage 0 to II cancers (>90%) and 72% for stage III, but it drops to 14% for stage IV, the stage of dissemination to distant organs [21]. Survival also depends on CRC phenotype since patients with MSI tumors have a better prognosis than patients with MSS tumors [22,23]. Other studies have been performed using meta-analyses on transcriptomic data to propose a consensus molecular classification (CMS) of CRCs by defining four subtypes that have been associated with a prognostic value for patient survival [24]. The CMS classification has an important prognostic value and indicates that in non-metastatic CRC (stages 0 to III), the prognosis is favorable for tumors in the CMS-1 subgroup and to a lesser extent for the CMS-2 subgroup. Conversely, in a metastatic situation (stage IV), it is the CMS-1 subgroup that is linked to the worst prognosis since the overall survival of patients with a CMS-1 tumor was 14.8 months against 31.9 months for CMS-2 tumors [25].

Therapeutically, tumor resection remains the primary treatment for all stages of the disease. However, for stages with lymph node involvement or distant metastases (in the liver and lungs), chemotherapy combined or not, with targeted therapy is proposed. Note that therapy using cetuximab or panitumumab, two anti-EGFR (Epidermal Growth Factor Receptor) antibodies, is proposed only to treat CRCs with wild-type KRAS [26]. In recent years, an increasing number of studies have focused on immunotherapy. The basis of immunotherapy is to overcome the mechanisms involved in immune tolerance to tumor self-antigens and to block the immunosuppressive response that occurs in the tumor microenvironment. This process is primarily driven by the inactivation and depletion of T cells via the activation of immune checkpoint inhibitors (ICIs) on the surface of T cells, which prevent them from recognizing tumor neoantigens. Current therapies target the PD-1 receptor (Programmed cell Death protein 1) and its ligand PD-L1 (Programmed cell Death protein Ligand 1), and CTLA-4 (Cytotoxic T lymphocyte Antigen 4).

In the exploratory NICHE study (ClinicalTrials.gov: NCT03026140), patients with early-stage MSS or MSI CRC and neoadjuvant treatment with a single dose of anti-CTLA-4 (ipilimumab) and two doses of anti-PD1 (nivolumab) led to 100% and 27% response in MSI and MMS tumors, respectively [27]. Several phase II and III randomized controlled trials are underway to evaluate the efficacy of immunotherapy in metastatic CRC of both phenotypes (first-line or refractory), with/without chemotherapy [28]. Once it will be validated in larger cohorts and with at least 3 years of recurrence-free survival data, neoadjuvant immunotherapy could potentially become the standard of care for a defined group of patients.

## 2. CXCL12 and Its Two Receptors CXCR4 and CXCR7

Chemokines are a group of small proteins of 8 to 12 kDa from the family of chemoattractant cytokines [29,30]. To date, about fifty chemokines have been identified, and they are structurally classified into subfamilies of chemokines C, CC, CXC and CX3C according to the presence of the “chemokine domain”, represented by the location of four cysteine residues conserved in the N-terminal domain necessary for the formation of disulfide bridges [29,30]. These proteins exert their function by binding to receptors with seven transmembrane domains, which are related to rhodopsin receptors [31]. Thus, there is the CR, CCR, CXCR and CX3CR receptor subfamily. Within each group, several chemokines can bind to several receptors, and inversely, one receptor can bind several chemokines. Because of this redundancy, the absence of chemokines or their receptors by gene invalidation of chemokines or their receptors does not necessarily lead to major effects, except for CXCL12 and its two receptors CXCR4 and CXCR7. Mice invalidated for each of these three proteins die during the embryonic or postnatal period, demonstrating the essential role of these proteins during embryogenesis [32,33,34].

The chemokine–receptor interaction was initially described to induce lymphocyte migration and recruitment [35,36]. However, it is now clear that their activity extends beyond immune cell migration. Numerous studies have documented that chemokine signaling also guides the migration of neurons, neural crest cells and germ cells during embryonic development and regulates the patterning and remodeling of the vascular system [37,38,39]. The chemokine–receptor is also a factor in inflammatory diseases [36,40,41], infections [30,40,41] and cancers [42,43,44]. One of the most studied chemokines is CXCL12, which exerts its biological functions by activating the two receptors CXCR4 and CXCR7.

## 3. Physiological Roles of CXCL12 and Its Two Receptors CXCR4 and CXCR7

### 3.1. Chemokine CXCL12

The chemokine CXCL12, also known as stromal-cell-derived factor 1 of the bone marrow (SDF-1), was originally discovered as a factor stimulating the growth of pre-B lymphocyte progenitors CD34+ (pre-B CD34+) [33,35,45,46] and is mainly responsible for the homing and maintenance of hematopoietic stem cells in the bone marrow.

CXCL12 is a homeostatic chemokine whose expression is constitutive in a wide range of tissues and organs such as bone marrow, liver, lung, heart, brain, spleen, and intestine [35,47]; however, its expression can be induced during inflammatory conditions [48,49]. It is expressed in human and mouse with a highly conserved structure, and the gene undergoes splicing that generates six isoforms (CXCL12α to ϕ), the alpha and beta forms being the predominant and ubiquitously expressed forms [50,51].

In the intestinal epithelium, CXCL12 is expressed in an increasing gradient of concentration from the base to the crypt surface [52]. This high expression at the crypt surface contributes to the constant turnover of epithelial tissue as the CXCL12-CXCR4 signaling axis stimulates intestinal epithelial cell migration and enhances the integrity of the innate barrier of the intestinal mucosal epithelium [53].

### 3.2. CXCR4 Receptor

The CXCR4 receptor (C-X-C motif receptor 4) was originally discovered as a co-receptor for HIV entry into lymphocytes [54]. Human (352 amino acids) and murine (359 amino acids) CXCR4 receptors share 89% homology and are ubiquitously expressed in both embryonic and adult tissue [55]. As the first receptor that can bind CXCL12, it was considered for a long time as its only receptor, since mice deficient in CXCL12 or CXCR4 have similar phenotypes with abnormalities in hematopoiesis, blood vessel formation in the gastrointestinal tract, cerebellar development, cardiac ventricular septum formation and significant embryonic lethality [32,33,56]. The monogamy relationship between CXCL12 and CXCR4 was disproved by the discovery of the orphan receptor RDC1, identified by cDNA cloning in the dog thyroid [57,58].

The interaction of CXCR4 with its ligand CXCL12 activates downstream signaling pathways, including Ras-MAPK, PI3K-AKT-mTOR, Jak2/3-STAT2/4, PLC β and γ2, NF-κB, and JNK/p38 MAPK via interaction with Gβγ subunits, while inhibiting adenylate cyclase and cAMP formation via interaction with Gαi [59]. This signaling pathways activation leads to an alteration in the expression of genes that will modulate different cellular functions such as actin polymerization, cell skeleton rearrangement or cell migration [60,61]. The physiological functions of CXCR4 are not only critical for development and homeostasis but also for the survival of cancer cells.

### 3.3. CXCR7 Receptor

More recently, another receptor CXCR7 (C-X-C motif receptor 7), renamed ACKR3 (Atypical Chemokine Receptor 3) in 2014, has been described to bind CXCL12 with 10-fold higher affinity than CXCR4 [62] and can also bind with CXCL11. A particularity is that the CXCL12-ACKR3 complex does not couple to a G protein but through activation of the β-arrestin pathway [63]. However, a study by Nguyen et al. in HEK293 cells shows that binding of CXCL12 to CXCR7 does not result in activation of signaling pathways via Gαi subunits but activates G-protein-coupled receptor kinase 2 (GRK2) via βγ subunits and phosphorylation of the receptor by recruitment of β-arrestin 2 [64]. In contrast to CXCR4, CXCR7 internalization occurs even in the absence of ligand binding and does not lead to receptor degradation [65].

Similar to CXCR4, CXCR7 can activate many intracellular signaling pathways, including AKT and MAPK pathways, via β-arrestins [63]. CXCR7, which does not activate calcium responses in the presence of CXCL12, is able to modulate CXCR4-activated calcium signaling through the formation of CXCR4/CXCR7 heterodimers [65,66,67], which can form in the absence of CXCL12 ligand [65]. However, contradictory data indicate that the activation of such heterodimers by CXCL12 leads either to a potentiation of the calcium response with a loss of early activation of ERK kinase [65], or conversely, to a decrease in this calcium response [67].

The physiological implications of the CXCR7 receptor have been demonstrated in CXCR7 knockout mice (CXCR7-/-), which die at birth due to abnormal heart valve development, highlighting the critical role of CXCR7 in cardiogenesis [68]. Other studies have shown that CXCR7 allows for the migration of central nervous system neurons during development by indirectly controlling their migration, through the regulation of the expression level of CXCR4, and the loss of CXCR7 function results in the production of neurons functionally deficient for both receptors [69].

The phenotypic differences described for CXCR4-/- and CXCR7-/- mice [32,34], and recent work examining the role of these receptors in zebrafish development [70,71], support the hypothesis that CXCR7 and CXCR4 have specific and distinct biological roles. In addition, several groups have established that CXCR7 acts as a “scavenger” or “decoy receptor” for extracellular CXCL12 but also for CXCL11, promoting constant cycling between the plasma membrane and the cytoplasm, and thus establishing a CXCL12 gradient. Thus, CXCR7 controls chemokine concentrations in the extracellular space, limiting signaling via other receptors [72,73]. According to a recent study, the balance between intracellular and membrane expression of CXCR7, and thus its scavenger function, is tightly regulated by CXCL12-induced phosphorylation of CXCR7 that ensures its subsequent protection against degradation [74]. This atypical function of CXCR7 is essential for the development of many organs, for the control and coordination of cell migration and positioning [75], and is not only dependent on CXCR7 but requires an intimate interaction between CXCL12, CXCR4 and CXCR7.

## 4. CXCL12/CXCR4/CXCR7: Pathological Role in CRC

Pathologically, chemokines and their receptors are involved in the development of infectious diseases, in particular the role of CXCR4 as a gateway for the HIV virus in CD4+ T cells [54]. However, recently, the involvement of chemokines has aroused a lot of interest in oncology [43,76,77]. The first evidence emerges from studies in breast cancer, with the involvement of CXCR4 in the control of metastatic dissemination [78].

### 4.1. Receptor Expression

Numerous studies have investigated the expression level of CXCR4 and CXCR7 receptors in solid cancers and in hematological cancers, given their involvement in the development of the hematopoietic system. These studies show elevated expression of one or both receptors in tumors compared to adjacent healthy tissues [79,80,81]. Furthermore, in CRC, Romain et al. showed that CXCR4 and CXCR7 expression increases with clinical stages [82]. Several authors have reported that receptor overexpression reflects disease progression and is therefore associated with tumor aggressiveness, decreased survival and poor prognosis [80,81,83,84,85,86,87]. Receptor expression is not only associated with tumor cells but also with endothelial cells of tumor microvessels [88,89] whether in colon, liver, pancreas, prostate, or lung cancers [90,91]. In contrast, Guillemot et al. described CXCR7 expression only in vessels of primary colorectal tumors and in liver and lung metastases [92].

### 4.2. CXCL12 Expression

In CRC, different expression patterns have been reported. The expression of CXCL12 can be increasing from healthy mucosa to adenomas and adenocarcinomas [93] or, on the contrary, decreasing [94]. Other studies show that CXCL12 expression is higher in tumors compared to healthy tissues [95], and still, others describe heterogeneous tumors since within the same cohort, some tumors express the chemokine strongly while others express it weakly or not at all [96,97]. In tumors, CXCL12 is expressed by epithelial cells but also by vascular endothelial cells [96] and stromal fibroblasts [97]. Finally, some studies observe no difference in expression between healthy mucosa and tumor [98]. In contrast, we showed that CXCL12 expression is strongly decreased in 94% of adenomas and 85% and 75% of MSI and MSS carcinomas, respectively [52]. Similarly, Wendt et al. describe an absence of CXCL12 expression in the CRC epithelium [99]. It is always difficult to understand the reasons underlying different levels of expression of a factor in the same cancer in different studies.

One of the reasons for these discrepancies could be the mixture of colon and rectal tumors in the cohorts and the fact that part of rectal tumors are either irradiated and/or chemically treated before resection, leading to changes in CXCL12 expression level [100,101]. Another reason might be the technique used. For instance, in immunohistochemistry, there may be differences in the reference of the antibody, its dilution, and in the unmasking and revelation technique (enzymatic, immunofluorescence). The heterogeneity of the tumor must also be taken into account, as analyses are usually performed on only a fragment of the tumor. Depending on how the samples are collected, it is possible to be in areas with high, low or no expression of the protein. The number of tissue sections must also be considered; with a limited number of sections, it is possible to be in a tumor area expressing or not the protein. For these reasons, it could be recommended to separately study rectal and colon tumors, as well as to combine the expression of the transcript with that of the protein since these two techniques request separate tumor samples [52].

### 4.3. CXCL12/CXCR4/CXCR7 Axis in Cellular Interactions

The interaction between tumor cells and the tumor microenvironment, which includes fibroblasts, immune cells and endothelial cells, participates to the development of tumor malignancy through the modulation of a wide variety of proteins in both cancer and stromal cells [102]. For example, there is bidirectional crosstalk between tumor cells and cancer-associated fibroblasts (CAFs). This crosstalk is mediated by cancer cells releasing factors that enhance the ability of fibroblasts to release various tumor-promoting chemokines, which in turn act on malignant cells to promote their proliferative, migratory and invasive properties. In this aspect, the CXCL12-CXCR4 pair plays a fundamental role in a large number of malignancies [103].

More specifically, mesenchymal stromal cells (MSCs) can be recruited to the stroma of developing tumors to enhance metastasis through their ability to secrete growth factors such as CXCL12 to promote tumor cell proliferation and tumor angiogenesis [104,105]. However, MSCs are also able to differentiate into CAFs by enhancing CXCR4 expression and activating the TGF (Tumor Growth Factor) pathway, therefore promoting growth and metastasis by secreting protumor factors [106]. Similarly, Todaro et al. showed that medium conditioned with fibroblasts isolated from primary colon tumors increases the clonogenicity of sphere-cultured colon cells and enhances the migration of CD44 stem cells isolated from CXCR4-expressing human tumors; this medium also converts non-migrating CD44v6-negative cells into migrating CD44v6-positive cells [107]. This phenotype can be mimicked by CXCL12, which also confers metastatic potential and a more aggressive phenotype to these progenitors in vivo.

MSCs present in the tumor stroma may also exert indirect pro-malignant actions by promoting tumor angiogenesis through the recruitment of endothelial progenitor cells and by facilitating the formation and maturation of the tumor vasculature [108]. These patterns are relevant in situations where the primary tumor expresses CXCL12.

In tumors not expressing CXCL12, other chemokines or growth factors (CCL4 or CCL5/CCL1 or CXCL8) released by CRCs have the ability to attract cells of the immune repertoire, angiogenic progenitors, and mesenchymal stem cells, resulting in a metastatic phenotype [109,110]; these molecules can also be produced by stromal cells [111]. In addition, MIF (Macrophage migration Inhibitory Factor) was shown to recruit MSCs to tumors by a physical interaction between MIF and CXCR4 expressing cells observed in vitro and in vivo [112]. Other factors such as fibroblast growth factor (bFGF), VEGF, platelet-derived growth factor (PDGF), insulin-like growth factor (IGF), and TGF-β have been further described for their contribution to tumor growth to MSCs [108].

Conversely, in the liver, hepatic stellate cells (HSCs) constitute the predominant population of CAFs, which are the main components of the tumor microenvironment [113]. Tumor/fibroblast interaction has been involved the progression of cancer, the CXCR4/CXCL12 chemokine axis being a main leader of malignancy [114]. In addition, HSCs, together with liver sinusoidal endothelial cells, are one of the principal sources of CXCL12 secretion in the liver, where they mediate not only the recruitment of CXCR4-expressing tumor cells, but also of CXCR4-expressing immune cells [114]. Immunohistochemical analysis of human liver show that the sinusoidal endothelial cells lining the hepatic vessel wall abundantly express the CXCL12 protein, which is therefore perfectly positioned to interact with circulating tumor cells for the formation of metastases [115].

Therefore, CXCL12 promotes communication between cancer cells and the surrounding non-neoplastic cells in the tumor microenvironment, including endothelial cells and fibroblasts, through activation of CXCR4 and CXCR7. The hypoxic tumor microenvironment can favor the upregulation of CXCR4 and CXCL12 in several cell types such as endothelial cells and cancer cells through mobilization of the hypoxia induced factor 1 (HIF-1α).

Concerning CXCR7-expressing cells, Guillemot et al. found that, in the primary CRC, the presence of the CXCR7 protein was restricted to tumor-associated endothelial cells, whereas it was absent in tumor cells [92]. However, others described CXCR7 expression in tumor-associated blood vessels but also by the malignant cells in CRC [82,116] and other cancer types [117,118].

We could speculate that CXCR7 expression in tumor vessels is a common feature of all cancers, whereas the presence of this receptor in malignant cells would be restricted to a particular type of cancer.

## 5. Prognostic Value of CXCL121/CXCR4/CXCR7 Axis

### 5.1. CXCL12 as a Prognostic Factor

Clinically, there are divergent viewpoints on the prognostic value of CXCL12 expression level. High expression is significantly associated with high tumor stage, lymphatic invasion, venous invasion, lymph node and distant metastases, and decreased survival [93,96,97]. Likewise, other studies suggest an association between CXCL12/CXCR4 expression and the induction of adenomas, carcinomas, and the development of metastases [94]. Transcriptomic analysis of a cohort of 49 CRCs and RNA-Seq data from TCGA for 375 CRCs indicate that increased CXCR4/CXCR7+CXCL12 signature expression is the only independent prognostic marker for the presence/occurrence of metastasis and decreased overall survival in both datasets [119].

In contrast, in two cohorts of 290 and 306 patients with stage III CRC, high cytoplasmic expression of CXCL12, assessed by in situ hybridization and immunohistochemistry, is associated with a better 5-year event-free survival [120]. Several studies, conversely, did not find a correlation between high CXCL12 expression levels and clinico-pathological parameters [88,121]. For example, in a meta-analysis of 25 articles published through 2017, increased transcript or protein expression of CXCL12 was not associated with TNM stage, age, gender, or diagnosis, but only with degree of tumor differentiation [121]. In another cohort of 444 CRCs with MSS phenotype [104], the two molecular subgroups C4 and C6 have higher levels of CXCL12 expression than the other four subgroups and are associated with a worse prognosis for patients [122].

Fushimi et al. showed that overexpression of CXCL12 in the CT26 syngeneic colorectal cell line in Balb/C mice resulted in an accumulation of dendritic cells and CD8+ T cells, which significantly slowed tumor growth after subcutaneous implantation [123]. A significant number of studies have shown that CD8+ T-cell infiltration of a tumor is associated with a better prognosis in CRC [124,125,126,127]. In a study of 613 stage III CRC specimens, high CD8+ T cell infiltration combined with high CXCL12 expression is associated with superior 5-year overall survival compared to patients with tumors with high CD8+ T cell expression alone [128].

In order to address these conflicting results, it can be hypothesized that during the early stages of carcinogenesis, CXCL12 production might participate in the transformation of the colonic mucosa at the beginning of the carcinogenesis process, whereas at later stages, a lower expression would avoid the recruitment of cytotoxic lymphocytes and facilitate the development of metastasis. Wendt et al. reported that tumor cells that do not express endogenous CXCL12 respond better to exogenous CXCL12 produced by distant organs, leading to metastasis in mice [99].

### 5.2. CXCR4 as a Prognostic Factor

Regarding the prognostic implication of CXCR4, the literature agrees that high CXCR4 expression in CRC patients is unfavorable, as it correlates with advanced tumor stage and increased risk of recurrence and distant metastasis [96,121,122]. Several meta-analyses conclude that there is a significant association between high CXCR4 expression and poor overall survival [80,121,129,130,131]. In a similar way, a recent study indicated a particularly poor prognosis for patients having CRCs jointly and strongly express CXCR4 and VEGF (Vascular Endothelial Growth Factor) in more than 50% of cells, and this combination of high expression is a strong and independent predictor of early distant relapse [132]. In another cohort, the CXCR4+CXCR7+CXCL12-β+ signature stratifies patients with risk of metastasis and in a TCGA dataset (n = 375), this signature predicts the presence of metastasis and overall survival [119]. Consistent with these observations, low CXCR4 expression in resections of CRC liver metastases is independently associated with a lower overall recurrence rate and thus improved disease-related survival [133].

In tumor–stromal cell interactions, CXCR4 and CXCL12 form an important signaling axis, with the interaction influencing adhesion, migration and invasion, reflecting the strong association of CXCR4 with the development of metastasis. In addition to being a prognostic biomarker, these findings are of clinical relevance given the emergence of new drugs targeting the CXCR4 receptor. In the context of a combination of molecular alterations, patients whose tumors overexpress CXCR4 and express the mutated KRAS gene have the worst prognosis [134,135].

Nevertheless, some studies describe the absence of significant correlation between CXCR4 expression and metastasis development. For example, Nagasawa et al., by multivariate regression analysis, found no significant association between CXCR4 transcript expression and a clinico-pathological factor in a cohort of 200 patients with CRC [136]. In the same way, work on a small cohort of liver metastases from CRC identified no difference in the level of CXCR4 expression between tumor tissue and adjacent healthy tissue [137]. Finally, Xu et al. observed that the level of CXCR4 expression in the center of tumors is not predictive of a poor prognosis, but instead its expression at the invasive border is [138].

### 5.3. CXCR4 as Stem Cell Marker

The following markers are considered markers of CRC stem cells (CSCs): CD133, CD144, CD24, CD166, CD44, CD29, ALDH1, LGR5, and emerging studies have also reported the involvement of the CXCL12/CXCR4 axis in several adult stem cells [131]. CD133 is one of the markers described to identify tumor-initiating cells (TICs) in several cancers and in colon cancer; it has been used to isolate CSCs [139,140]. However, CD133 expression is not only limited to CSCs [141,142], and in order to identify these cells more accurately, additional markers have been considered. This is the case, for example, in the study by Zhang et al. who demonstrated that CXCR4 expression could be used in addition to CD133 expression to characterize colorectal CSCs [143]. In addition, a high percentage of double-positive cells for these two markers in human CRCs positively correlates with the presence of lymph node metastases [144]. Another example has been described where Lgr5+/CXCR4+ colonic cancer cells respond to the properties of CSCs through a greater ability to form spheres in vitro, develop tumors in vivo and resist chemotherapy. Furthermore, high levels of Lgr5 and CXCR4 expression in resected human CRCs correlate with poor prognosis [145].

The following markers are considered markers of CRC stem cells (CSCs): CD133, CD144, CD24, CD166, CD44, CD29, ALDH1, LGR5, and emerging studies have also reported the involvement of the CXCL12/CXCR4 axis in several adult stem cells [131]. CD133 is one of the markers described to identify tumor-initiating cells (TICs) in several cancers and in colon cancer; it has been used to isolate CSCs [139,140]. However, CD133 expression is not only limited to CSCs [141,142], and in order to identify these cells more accurately, additional markers have been considered. This is the case, for example, in the study by Zhang et al. who demonstrated that CXCR4 expression could be used in addition to CD133 expression to characterize colorectal CSCs [143]. In addition, a high percentage of double-positive cells for these two markers in human CRCs positively correlates with the presence of lymph node metastases [144]. Another example has been described where Lgr5+/CXCR4+ colonic cancer cells respond to the properties of CSCs through a greater ability to form spheres in vitro, develop tumors in vivo and resist chemotherapy. Furthermore, high levels of Lgr5 and CXCR4 expression in resected human CRCs correlate with poor prognosis [145].

### 5.4. CXCR7 as a Prognostic Factor

Since its discovery in 2005 [62], the role of CXCR7 in the carcinogenesis of many cancers has been well documented, and it is expressed in a wide variety of cancers and tumor-associated blood vessels, including colon, liver, pancreatic, prostate and lung cancers [83,146]. There are conflicting observations regarding the role of CXCR7 in the nature of the site of metastasis development. The expression of CXCR7 and CXCL12 is higher in lung metastases than in primary CRC, whereas the expression of CXCR4 in both sites is not statistically different [147]. Previous studies have observed that CXCR4 expression is higher in liver metastases than in primary CRC tumor tissue [148,149] and suggest that the mechanism of development of liver and lung metastases is different. This agrees with the in vivo experience of Guillemot et al., who showed that CXCR7 is a key factor in the progression of CRC metastases specifically in the lungs, since systemic treatment of mice with CXCR7 antagonists reduces metastasis in the lungs but not in the liver, after intravenous injection of HT-29 or C26 cells expressing CXCR7 [92].

In the study by Yang et al., positive CXCR7 expression is associated with the presence of lymph node metastases, distant metastases and advanced TNM stage [85]. Sherif et al. significantly observed cytoplasmic expression of CXCR7 in 11% of colorectal adenomas and 72.4% of CRC [150]. In contrast to studies favoring a poor prognosis for high CXCR4 and CXCR7 expression in CRC, Kheirelseid et al. observe that patients with above-median expression have lower mortality (mean survival 46 months) than patients with below-median CXCR7 expression (mean survival 27 months). Similarly, lower expression of CXCR4/CXCR7 and CXCL12 is associated with increased tumor size, local invasion, poor differentiation, advanced lymph node stage, advanced tumor stage, and lymphovascular invasion [151].

Therefore, although the expression level of CXCL12, CXCR4 and CXCR7 has been considered a prognostic factor in several human tumor types (Table 1), none of the actors of this axis have yet been definitively validated as pro-tumoral factors. Studies suggest that the CXCL12 axis is a promoter rather than a tumor initiator.

**Table 1:** Clinical significance of CXCL12, CXCR4 and CXCR7 expression levels in CRC.

Authors	CXCL12		CXCR4		CXCR7		References
	Expression	Prognosis	Expression	Prognosis	Expression	Prognosis	
Romain, 2017	↓; ↓	If ↑; ↓ OS					[52]
Fan (meta-analysis), 2018	-				↑	↓ OS; ↓ DFS	[81]
Romain, 2014	-		↑		↑		[82]
Kim, 2005; 2006	-		↑	↓ OS			[84,85,86,87,88,89,90,91,92,93,94,95,96,97,98,99,100,101,102,103,104,105,106,107,108,109,110,111,112,113,114,115,116,117,118,119,120,121]
Yang, 2015	-				↑	↓ OS; ↓ DFS	[85]
Yang, 2015	-				↑	↓ OS; ↓ PFS	[85]
Xu, 2018	-		↑	↓ OS			[86]
Ingold, 2009	↑		vascular	↓ OS			[88]
Guillemot, 2012	↑		↑		↑		[92]
Greijer, 2008	↑						[93]
Frick, 2011	↓		↑				[94]
Amara, 2015	↑	↓ OS	↑	↓ OS			[95]
Yoshitake, 2008	If ↑	↓ OS	If ↑	↓ OS			[96]
Akishima-Fukasawa, 2009	If ↑	↓ OS					[97]
Mousavi, 2018	→	→	→	→			[98]
Wendt, 2006	↓						[99]
Mitchell, 2019	↑	↓ OS	↑	↓ OS	↑	↓ OS	[111]
Stanisavljević, 2016	↓; ↑	↓ DFS; ↑ DFS	↑	stage III, ↓ DFS			[112]
Li (meta-analysis), 2017	↑	↓ OS; ↓ DFS	↑	↓ OS; ↓ DFS			[113]
Lalos, 2021	↑	↑ OS					[120]
Schimanski, 2005	-		If ↑	↓ OS			[122]
Lv, 2014	-		↑	↓ OS; ↓ DFS			[123]
Li, 2015			↑	↓ OS			[122]
Jiang, 2019	-		↑	↓ OS			[125]
Ottaiano, 2020	-		↑	↓ OS			[124]
Yopp, 2012	↓; ↑	→	If ↑	↓ OS; ↓ DFS			[127]
Nagasawa, 2021	-		→	→			[128]
Jiao (CRC liver metastases), 2019	→	→	→	→			[129]
Xu, 2007	-		↑ invasive border	↓ OS			[132]
Kheirelseid, 2013	-				If ↑	↑ OS	[144]

↑: upregulated; ↓: downregulated; →: no change; -: not evaluated; DFS: disease-free survival; PFS: progression-free survival.

## 6. Mechanisms of Expression Regulation

### 6.1. Regulation of CXCL12 Expression

For both overexpression and loss of CXCL12 expression, several molecular mechanisms have been proposed. Intratumoral hypoxia has been shown to be a factor that promotes the overexpression of CXCL12 in vivo [152,153,154], ex vivo [155] and in vitro [153,155]. In these studies, CXCL12 expression is associated with hypoxic or HIF-1α-expressing areas and this association has been confirmed using siRNAs directed against HIF-1α [93,152,153,154]. In endothelial cells and under hypoxic conditions, the hypoxia-induced upregulation of CXCL12 expression was clearly attributed to the direct binding of HIF-1α to its specific binding sites on the CXCL12 promoter [153].

Moreover, several mechanisms have been proposed to explain the loss of CXCL12 expression. Hypermethylation of the CXCL12 promoter in CRCs has been proposed by Wendt et al. [99], as well as in cervical tumor lines and biopsies, observed by Yadav et al. [156]. In our study of a cohort of 444 MSS CRCs, we showed that the CpG islands of the CXCL12 promoter are methylated in only 30% of tumors [82]. In the same studies, we also reported that, in vitro, treatment of three colonic lines with histone deacetylases (HDAC) inhibitors such as butyrate and valproate restored CXCL12 expression and increased acetylation of histone H3 of the CXCL12 promoter [52]. In vivo, valproate treatment of APC mutant mice (APC^Min/+^) decreases the number of intestinal tumors and slows down tumor growth in ectopic xenografts while restoring CXCL12 expression [52]. In these CRCs tissues, an analysis of the expression of 85 genes regulating epigenetic processes showed a loss of expression of a histone acetyltransferase, the protein P300/CBP-associated factor (PCAF), and forced expression of PCAF in colon cancer cell lines restored the expression of CXCL12 [52]. A further study in the blood–brain barrier, with endothelial cells lacking CXCL12 expression and pericytes expressing it, shows that the CXCL12 promoter is not methylated in both cell types; in contrast, ChIP experiments indicate reduced levels of histone acetylation of the promoter in endothelial cells compared with pericytes [157]. It is well documented that histone deacetylation of promoters generates a compact chromatin configuration that renders chromatin inaccessible to transcriptional factors and induces transcriptional repression [158]. Therefore, histone acetylation changes/defects associated with methylation of the CXCL12 promoter in some CRC subtypes would be involved in CXCL12 expression changes [52].

Functionally, cells with a decrease/loss in CXCL12 expression would be likely to be attracted to tissues expressing CXCL12, such as metastasis sites [47]. Moreover, this expression defect would contribute to the resistance to anoikis with, consequently, a migration and dissemination of tumor cells favoring the development of metastasis [159].

### 6.2. Regulation of CXCR4 Expression

The mechanisms leading to the high receptor expression are not clearly defined. A mode of regulation of gene expression is related to the intrinsic instability of transcripts due to the presence of adenylate-uridylate-rich element (AREs) in their 3′-UTRs, which are targeted by RNA-binding proteins for degradation, among which are those of cytokines or chemokines [160]. However, the role of these AREs may be compromised in cancer, largely due to a deficiency in proteins that promote mRNA degradation. These sequences were found in the 3′UTR of many labile mRNAs that encode proto-oncoproteins (c-myc, c-fos, c-jun) and cytokines [160]. Al-Souhibani et al. showed that in breast tumor cells, the CXCR4 gene harbors a functional ARE in its 3′-UTR portion, a potential target for the RNA degradation proteins, TTP and HuR [161]. They also demonstrate that overexpression of HuR combined with low expression of TTP results in increased stability of CXCR4 mRNA and consequently higher levels of protein that will promote detachment and migration of breast tumor cells to distant sites [161].

Tumor progression is associated with intratumor hypoxia, which leads to increased vascular density, and HIF-1α is a transcription factor that allows for adaptation of tumor cells to hypoxia [162]. In CRC, hypoxia has been shown to promote increased expression of CXCR4 [82,163], and in human colonic cell lines, this effect is mediated by the transcription factor HIF-1α [82]. Many studies have shown that HIF-1α is expressed at elevated levels in highly aggressive CRCs [164] and plays a major role in regulating the expression of many genes involved in angiogenesis and chemotaxis via the CXCL12/CXCR4 axis [165]. Our team has demonstrated that CXCR4 expression is increased by hypoxia in human colonic cell lines [82]. Additionally, the combined expression of CXCR4, HIF-1α and VEGF is strongly correlated with the presence of lymph node metastasis and distant metastasis in human CRC [166]. Zong et al. performed a bioinformatics analysis of Gene Expression Omnibus (GEO) data of HCT-116 cells subjected to acute and chronic hypoxia to identify genes differentially expressed in normoxic and hypoxic conditions. Among these genes, they found CXCR4 whose expression is upregulated under these conditions [163]. Numerous publications have reported the direct involvement of the two hypoxia-inducible factors, HIF-1α and HIF-2α, on the increase in CXCR4 expression [167], as the promoter of the gene encoding CXCR4 contains a hypoxia response element (HRE) [168,169].

Studies have suggested that the ERK1/2 and PI3K/Akt pathways, mediators of chemokine-induced migration, are activated by hypoxia in many cell types [170]. In addition, in vitro treatment of endothelial progenitor cells with specific inhibitors of the ERK1/2 or PI3K/Akt pathway indicates that only Akt activation is required for hypoxia-induced increase in CXCR4 expression and increased chemotaxis [171]. Other studies report that activation of the PI3K/Akt pathway can increase translation of HIF-1α-coding mRNA and stabilization of the protein under hypoxic conditions [172], which would promote increased CXCR4 expression. Likewise, reduction of CXCR4 expression by siRNA in human colonic tumor cells cultured in hypoxia decreases CXCL12-induced phosphorylation and activation of Akt, while ERK activation is unchanged [82].

Epigenetic alterations have also been described to regulate CXCR4 expression in CRC. MicroRNAs (miRNAs or miRs) have emerged as critical regulators of carcinogenesis and tumor progression and are described to modulate cell proliferation, apoptosis, invasion, angiogenesis, and metastasis [173]. It is now evident that certain miRNAs may be involved in the activation of the CXCL12/CXCR4 axis and thus participate in the progression of CRC to metastasis by controlling CXCR4 expression. For example, miR-9 expression is decreased in late-stage CRC and low miR-9 levels are significantly associated with lymph node metastasis [174]. Furthermore, Kaplan–Meier analysis reveals that decreased miR-9 expression is significantly correlated with shorter median survival time, suggesting that miR-9 is an independent prognostic marker for overall survival of CRC patients and acts as a potential tumor suppressor gene [174]. In the same study, the authors show that in vitro, this miR inhibits cell migration and invasion. Moreover, a bioinformatics analysis of miR-9 target genes identified CXCR4, whose transcript has a possible miR-9 binding element in its 3′-UTR region. Using a Dual-Reporter assay, this observation was validated by demonstrating that miR-9 negatively modulates the transcriptional and protein expression of CXCR4 by binding directly to its 3′-UTR. In vivo, injections of colonic tumor cells overexpressing miR-9 into the tail vein of mice resulted in fewer lung metastases than with control cells, a similar effect obtained with cells deleted for CXCR4 expression [174].

Another study investigated the prognostic value of miR-126 expression level associated with that of CXCR4 in CRC, and an inverse correlation was observed between miR-126 and CXCR4 protein expression in CRC [175]. Furthermore, low miR-126 and high CXCR4 expression is associated with distant metastasis, TNM clinical stage, and poor survival, Multivariate analysis indicates that miR-126 is an independent prognostic factor for overall survival [176]. In another study, the same team showed that miR-126 negatively regulates CXCR4 through the AKT and ERK1/2 signaling pathways, and thus this miR functions as a tumor suppressor in CRC cells [175].

Another miR might be involved in the regulation of CXCR4, miR-622, which is underexpressed in CRC metastases and has been described as a potential tumor suppressor gene by slowing down KRAS-dependent tumor and metastasis formation in mice [177]. By overexpressing KRAS in cells, the authors restore normal tumor growth. The same authors subsequently showed that in vitro, overexpression of miR-622 in HUVEC cells inhibits capillary tube formation and that in vivo, this overexpression in HT29 cells xenografted to mice, slows tumor growth by strongly decreasing angiogenesis [177]. In parallel, analyses also showed that CXCR4 and VEGF-α expression is strongly decreased in these tumors. Similarly, as for miR-9, miR-622 has a binding site in the 3′-UTR region of the CXCR4 transcript and can therefore directly inhibit CXCR4 expression. Since VEGF is a target of CXCR4, the anti-angiogenic impact of miR-622 can be mediated by the repression of CXCR4 and consequently, reduces VEGF expression [178].

MiR-133b has also been described as a regulator of CXCR4 expression, with its expression being much lower in metastatic CRCs (stages C and D) than in early tumors (stages A and B) [179]. Using bioinformatics algorithms to identify targets of this miRNA, several targets including CXCR4 have emerged from the analysis, and a luciferase assay showed the existence of a binding site for miR-133b in the 3′-UTR of the CXCR4 transcript [179], as had also been described for miR-9 [174], miR-622 [177] or miR-139 [180].

Finally, the relative expression of the CXCR4 transcript and protein are significantly suppressed by transfecting DLD-1 and SW480 colonic cells with miR-140-3p, and this effect is reinforced by the existence of a binding site of this miR on the CXCR4 messenger [181]. In another context, the human miR-125b has been described to positively regulate Wnt/β-catenin signaling by targeting APC expression; however, in a positive feedback, the increase in miR-125b in turn leads to increased expression of CXCR4 [182].

Studies also highlight the possibility of modulation of CXCR4 expression by changes in the DNA methylation profile and/or histones of the promoter. A recent work by Stuckel et al. showed that the overexpression of CXCR4 in human CRCs is observed in both colonocytes and stromal cells. The authors found that this overexpression is not the result of hypermethylation of the CpG islands of the CXCR4 promoter but rather of an increase in 5-hydroxymethylcytosine (5hmC), a marker of active demethylation of a gene [183]; and in this case, the accumulation of 5hmC would reflect increased transcription of CXCR4 in the CRC [184]. This work complements other studies demonstrating the regulation of CXCR4 expression by epigenetic processes associated with genome methylation. Such 5hmC marks have been described for genomic and circulating DNA from different cancer types, including CRC, and were distributed in transcriptionally active regions. In addition, by using 5hmCs as biomarkers, it was possible to separate patients who developed CRC from those who did not, which also allowed the definition of marks to discriminate genomic DNA from tumor and healthy tissues [185].

In addition, studies carried out in vitro [186] and in vivo [187] show that cells lacking CXCR4 expression under stress conditions can begin to express the receptor. This is the case in Ewing’s sarcoma cell lines, in which the CXCR4 promoter is highly enriched in activating but also repressive histone marks. These cells, once under stress, show a loss of the repressive mark H3K27me3 while the activating mark H3K4me3 is increased with a consequent increase in the expression of CXCR4 [187].

Demonstrating that increased CXCR4 expression facilitates the development of liver but not lung metastases, and that decreased CXCR4 also reduces liver metastasis without affecting lung metastasis, Urosevic et al. also showed that transcription factors of the ETS family mediate CXCR4 expression downstream of RAS-ERK1/2 signaling. ETV4 and ETV5 factors induce a strong expression of CXCR4 in human colorectal lines [188]. It is also known that the deregulation of genes of the HOX family of transcription factors facilitates the progression of cancers through various mechanisms [189]. In two independent CRC cohorts, high HOXB5 expression was positively correlated with the presence of lymph node metastases, distant metastases, poor tumor differentiation and advanced clinical stage [190]. Moreover, overexpression of HOXB5 in the Caco-2 colorectal cell line leads to changes in the expression of several genes involved in metastasis, including CXCR4, and the use of reporter gene systems shows that CXCR4 is a transcriptional target of HOXB5 [190].

### 6.3. Regulation of CXCR7 Expression

While the literature provides numerous studies regarding the mechanisms of regulation of CXCR4 expression in CRC, much less data are available for CXCR7. Evidence for an impact of hypoxia and the transcription factors HIF-1 and -2 exists in other cell types, such as in bone marrow-derived mesenchymal stem cells where the PI3K/Akt-HIF-1α-CXCR4/CXCR7 pathway is essential for cell migration, adhesion, and survival [168] or in glioblastoma cells [191]. The only study published to date in CRC is a work by our team that showed that in human colonic cells, hypoxia or HIF-1α silencing does not alter the expression level of CXCR7 [82].

Gene expression can be regulated by transcription factors such as the HIC1 (Hypermethylated in Cancer 1), which is hypermethylated in many tumors including CRC [192,193], and inactivation following hypermethylation is thought to be a tumorigenesis-triggering event [194]. The search for HIC1 consensus binding sites (HiRE) in the CXCR7 regulatory region identified 11 putative HiREs to which HIC1 could bind directly [195]. HIC1 gene knockdown, CXCR7 promoter HiRE mutations and ChiP-seq approaches demonstrate that CXCR7 is a direct target of HIC1, which acts as a direct repressor of CXCR7 expression [195]. This suggests that in tumors with loss of HIC1 expression, the subsequent increase in CXCR7 may participate in tumor progression.

Although, similar to its partner CXCR4, CXCR7 expression can be regulated by epigenetic mechanisms involving miRs in different tumor types [195,196,197,198]; to date, no data in the literature have demonstrated the involvement of a miR to regulate CXCR7 expression in CRC.

## 7. Implication of CXCL12/CXCR4/CXCR7 Axis in Metastatic Dissemination

**Figure 1:**
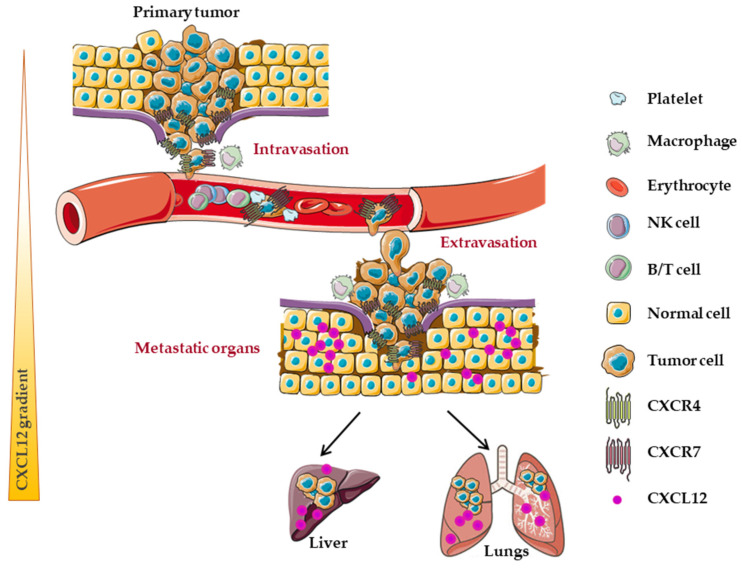
Process of metastatic dissemination in CRC. Tumor cells that no longer express CXCL12 will migrate to organs of metastasis via blood circulation. The expression of the CXCR4 and CXCR7 receptors allows for the intra- and extravasation of the cells through the vessels and then the implantation in the liver and the lungs where CXCL12 is strongly expressed. During intravasation of tumor cells into circulation, macrophages localized to perivascular areas within tumors help tumor cells traverse vessel barriers. In the circulation, platelets support tumor cell survival by protecting them from cytotoxic immune cell recognition.

For many years, the signaling mediated by this axis has been described to participate in the different aspects of tumor progression and dissemination (Figure 1). To better determine the respective involvement of each partner of this axis, different approaches have been used, such as interfering RNA, genetic editing by overexpression or loss of function, pharmacological inhibitors, neutralizing antibodies in vitro or in vivo. To understand the involvement of CXCL12 in tumor dissemination, it is necessary to separate the role of the chemokine itself from that of the CXCR4 and CXCR7 receptors, as well as the level of expression of CXCL12 in the primary tumor and the sites of metastatic implantation where it is highly expressed [52,78].

One hypothesis is that before metastasis develops, many CRC cells undergo DNA hypermethylation on the CXCL12 promoter [99], such that autocrine and paracrine CXCL12 signaling is reduced and tumor cells can migrate along a gradient that leads them to distant organs, known to highly express the chemokine [47]. This process would be initiated early in colonic carcinogenesis since CXCL12 expression is already lost at the adenoma stage [52]. This downregulation of CXCL12 expression also prevents colonic tumor cells from undergoing anoikis, a form of apoptosis when cell–cell contact is lost between epithelial cells [159].

### 7.1. CXCL12

The implication of CXCL12 has been demonstrated in different models. For example, in the dorsal skinfold chamber model of syngenic BALB/c mice, Kollmar et al. studied the effects of increasing concentrations of CXCL12 on tumor growth and angiogenesis induced by CT26 cell implantation [199]. In vivo, CXCL12 accelerates tumor growth through induction of angiogenesis, cell proliferation and inhibition of apoptosis [199]. In another study, the same authors used the same experimental model but performed a hepatectomy in mice [200]. It is known that liver resection is associated with liver regeneration and a local and systemic release of potent growth factors, including chemokines [201,202]. This model permits to understand the role of CXCL12 on the dissemination of CT26 cells in the tissues around the skinfold chamber. The authors report that neutralization of CXCL12 with an antibody promotes tumor extension to nearby tissues, accelerates angiogenesis and neovascularization, increases VEGF expression, microvascular permeability and increases CXCR7 expression [199]. Moreover, neovascularization and tumor growth are reduced after CXCR4 neutralizing treatment. Therefore, in the absence of CXCL12, signaling by CXCR4 is interrupted and an alternative pathway must be considered that would be carried by CXCR7. CXCR7 has been described to increase the production of VEGF [182], which would be the trigger of the pro-angiogenic effect observed after neutralization of CXCL12 [199].

Conversely, CXCL12 has also been described as an anti-tumor molecule in pancreatic cancer [203]. In CRC, Wendt et al. described a strong decrease in CXCL12 expression [99] and when colon cells treated with a demethylation agent to restore CXCL12, are injected into the tail vein of mice, metastatic tumor formation is greatly reduced as compared to cells lacking CXCL12. A similar situation has been observed in APC mutant mice that spontaneously develop CRCs. When these mice are treated with a histone deacetylase inhibitor, such as valproate, there is a re-expression of CXCL12 and a decrease in the number of tumors [52].

### 7.2. CXCL12 and CXCR4

The contribution of CXCR4 in tumor cell migration involves several cellular aspects that all converge toward the facilitation of cell migration and invasion. For example, overexpression of CXCR4 promotes the formation of pseudopodia through actin polymerization [78] and reorganization of the cytoskeleton [204]. Other processes are induced under hypoxic conditions such as epithelial–mesenchymal transition (EMT) and overexpression of α2, α5 and β1 integrins [205].

Migration and invasion processes also involve proteolytic activities induced by the important secretion of gelatinases such as MMP-2, MMP-9 or matrylisin-1 (MMP-7) [204,206,207]. One study showed that the ability of CXCL12-mediated cell migration and invasion is highly dependent on MMP-9 secretion and activity via Akt and ERK/MAPK signaling [204] and on β-catenin translocation in the nucleus, suggesting the interaction of the CXCL12/CXCR4 axis with the Wnt-β-catenin signaling [206].

At the signaling level, CXCR4 regulates the migratory and invasive ability of cells via the MAPK/ERK1/2 and PI3K/Akt signaling pathways activated by CXCL12-CXCR4 binding [82,204,208,209]. Similarly, CXCL12 binding to CXCR4 activates pro-metastatic signaling by decreasing E-cadherin expression but inducing ICAM-1 expression (InterCellular Adhesion Molecule) [210]; however, it has been shown that high levels of ICAM-1 in CRCs are associated with decreased tumor progression and liver metastasis [211]. Activation of the CXCR4/CXCL12 axis also involves the TGF-β pathway to promote invasion, angiogenesis, and promotion of distant metastasis by promoting differentiation of hepatic stellate cells into CAFs [212]. Furthermore, CXCR4 knockdown strongly reduces in vivo tumor growth associated with the reduction of tumor capillaries and intra-tumoral blood flow without affecting VEGF expression [213]. Moreover, in HUVEC cells, CXCR4 knockdown strongly inhibits angiogenesis after stimulation with CXCL12 ligand, by reducing EGFR, VEGF, and MMP-2, affecting MAPK/ERK, PI3K/Akt and Wnt/β-catenin pathways [207]. Another study showed that CXCL12 could stimulate the metastatic behavior of colonic cells expressing CXCR4 by increasing cell proliferation and adhesion to fibronectin [214]. These studies agree with those of Gouveia-Fernandes et al. who show that overexpression of fibronectin confers invasive and disseminative potential to HCT15 cells by promoting activation of the CXCL12/CXCR4 axis through modulation of α3 and β3 integrin expression [215].

Furthermore, Zeelenberg et al. demonstrate that CXCR4 expression is regulated positively by the tumor microenvironment, but it appears that CXCR4 is not required for tumor cell entry into metastatic sites, but rather for the establishment of micro-metastases [216]. Mice in which murine CT-26 colonic cells deficient in CXCR4 by retention of the receptor at the level of the endoplasmic reticulum, were injected into the spleen or the tail vein, indicate that CXCR4 would not play a role in invasion, but rather in the survival of the cells to form micro-metastases without impacting their proliferation [216].

In the same idea, Matsusue et al. showed that HCT116 cells stimulated by CXCL12 become resistant to apoptosis, and the use of AMD3100 reduces this CXCL12-dependent anti-apoptotic ability [217]. In vivo, these cells metastasize because CXCR4-positive cancer cells selectively survive by an anti-apoptotic effect and by the secretion of CXCL12 by stellate liver cells. Thus, these liver cells, under the action of TFG-β1 secreted by the tumor cells, will differentiate into CAFs [217]. The involvement of CXCR4 in the metastatic process could also be potentiated by another receptor, CXCR3 [218], which is activated by the chemokine CXCL10 by inducing cytoskeletal rearrangements, migration, invasion, expression of the matrix metalloproteinase MMP-2/9, and cell survival through the activation of ERK1/2, Akt and protein kinase G [219]. These ideas are supported by the findings of Tan et al. who propose that overexpression of CXCR4 by tumor cells in the hepatic metastatic microenvironment stimulates the production of CXCL12 by stellate cells, which through a paracrine action, stimulates the secretion of TGF-β1 by tumor cells, necessary for the differentiation of hepatic stellate cells into CAFs [212]. These studies suggest that modulation of the CXCL12-CXCR4 interaction can have a strong impact on tumor dissemination to target organs.

### 7.3. CXCL12 and CXCR7

A growing number of studies are emerging to understand the mechanisms by which CXCR7 participates in the growth and progression of colon cancer to organs of metastasis. Thus, the involvement of CXCR7 in colorectal tumorigenesis has been discussed in several models and is through the regulation of proliferation, survival, migration, invasion, angiogenesis, tumor growth and metastatic dissemination [89,218]. CXCR7 gene silencing represses cell proliferation and invasion and induces apoptosis with decreased expression of p-ERK, β-arrestin, PCNA and MMP-2 but with increased expression of caspase-3 [220]. Subcutaneous tumors induced by SW480 cells deleted for CXCR7 expression are significantly smaller than those in control groups [220]. CXCR7, but not CXCR4, expression can be increased by lipopolysaccharide treatment in cells expressing both TLR4 and the MD2 coreceptor [221]; additionally, in patients, high expression of TLR4, MD2 and CXCR7 is associated with tumor cell infiltration in lymph nodes and distant metastases [221].

The involvement of CXCR7 has also been described in transendothelial migration, and CXCR7 expression is found both in tumor cells and in tumor-associated vessels. However, using the endothelial cell line HUVEC, it was shown that this expression by vessels is not necessary for CXCL12-mediated transendothelial migration, and this process requires CXCR7 expressed by tumor cells, without involving CXCR4 [222]. CXCR7 appears to have angiogenic activity since its overexpression in colonic cells cultured with HUVEC cells promotes the formation of capillary tubes, and the stable extinction of CXCR7 in colonic cells prevents this tube formation [89]. CXCR7 exhibits low levels of expression in normal mature vascular endothelial cells but is highly expressed in endothelial cells of neovascularized tumors [223]. This effect might be a consequence of the stimulation by CXCR7 of VEGF production by endothelial cells via activation of the ERK and AKT pathways [89].

An in vivo study with transgenic mice overexpressing CXCR7 in the intestine showed that this overexpression exacerbates DSS treatment-induced inflammation by causing extensive infiltration of myeloid suppressor cells, M2-like macrophages, and Tregs in the colon, associated with elevated amounts of the proinflammatory cytokines TNF-α, IL-6, and c-Myc but decreased numbers of CD8+ T cells [205]. This CXCR7 overexpression also increases tumorigenesis in APC^Min/+^ mice and these effects are amplified when mice overexpress the CXCR4/CXCR7 heterodimer [224].

Although the implication of CXCR7 in the metastatic process is well demonstrated, there are still some questions about its capacity to direct the dissemination more specifically in an organ. Guillemot et al. have shown that in mice, systemic treatment with specific CXCR7 antagonists prevents the dissemination of cells in the lungs but not in the liver [92]. Concomitantly, higher expression of CXCL12 and CXCL11 was found in tumor areas in the lung compared with the liver, indicating that distinct pathways regulate the mechanism of pulmonary and hepatic metastatic spread. In another study in human CRCs, CXCR7 expression was also found to be higher in lung metastases than in the primary tumor [138].

### 7.4. CXCL12, CXCR4 and CXCR7

For a possible mechanism of action of CXCL12 in promoting metastasis, numerous works have highlighted the role of matrix metalloproteinases (MMPs), proteinases responsible for degradation and remodeling of the extracellular matrix (ECM). Thus, the persistent localization of these enzymes at the interface between migrating CRC cells and the surrounding stroma has been demonstrated, supporting a role for MMPs in CRC invasion and metastasis [225]. This study shows that none of the three CRC cell lines tested express MMP-2 or MMP-9. In contrast, subcutaneous tumors induced by transplantation of these cells express limited amounts of MMP-2 and MMP-9 while caecal tumors express them in large amounts [225] showing the role of murine stromal cells in the production of these proteinases.

Similarly, in myeloma cells, CXCL12 induces the expression of matrix metalloproteinases (MMPs) such as MMP-9, membrane MMPs such as MT1-MMP, represses the expression of inhibitors such as TIMP-1, promoting cell invasion in vitro [226]. However, these observations do not support a possible role for CXCL12 in the invasiveness of colonic tumor cells that no longer express CXCL12 [53,123]. In addition, it is possible to speculate that in vivo, colonic tumor cells acquire the ability to produce MMP regulatory factors other than CXCL12, such as mutations in tumor suppressor genes or proto-oncogenes, changes in the microenvironment, extracellular matrix composition, tissue oxygenation and inflammation [227].

## 8. Targeting of the CXCL12/CXCR4/CXCR7 Axis in CRC

### 8.1. Preclinical Studies

A recent report found that CXCL12 and relative expression of the CXCL12-CXCR4 axis are independent prognostic factors for 5-year relapse-free survival [120]. Multiple preclinical studies have evaluated the efficacy of many agents; however, only a few drugs targeting this axis have been approved for clinical use. These agents include anti-CXCR4 neutralizing antibodies, interfering RNAs or antagonist molecules targeting CXCR4 or CXCR7, or small peptides specifically blocking CXCR4 (Table 2).

In the clinic, the molecules used mainly target CXCR4, and a molecule more specifically targets CXCL12 [228]. Many preclinical studies targeting the CXCL12/ CXCR4/CXCR7 axis have been published, but few have focused on CRC.

### 8.2. AMD3100

The best-known molecule to inhibit the biological effect of CXCR4 is the molecule commonly known as AMD3100 or plerixafor (Mozobil). The Food and Drug Administration (FDA) approved AMD3100 in 2008 for use in the mobilization of hematopoietic stem cells for transplantation in patients with non-Hodgkin’s lymphoma [229,230]. AMD3100 is a specific antagonist of CXCR4 of the bicyclam family [231]. This drug acts as an antagonist by binding to one glutamine and two aspartate residues in the CXCR4 receptor, preventing the conformational change necessary to activate intracellular kinases [232]. It is the most frequently used drug in clinical trials targeting the CXCL12-CXCR4/CXCR7 axis and has been described in several studies in hematological, breast, pancreatic, lung cancer [231,233].

In an orthotopic model of liver metastasis using the murine colonic line C26, blocking CXCR4 with AMD3100 reduces the number and size of liver metastatic sites [234]. Immunohistochemical analyses revealed a significant decrease in the expression of α-SMA, a marker for hepatic stellate cells, in the liver foci of AMD3100-treated mice compared with control mice [234]. The promotion of VEGF production by stellate cells has been demonstrated in liver metastases in vivo [102], facilitating the recruitment of sinusoidal endothelial cells and the transition from avascular to vascular stage in these metastatic sites. In this context, a decrease in stellate cells induced by AMD3100 could therefore alter the angiogenic response and the blood supply of oxygen and nutrients to the tumor. However, AMD3100 has been described to also interact with CXCR7 but as an agonist [235]. AMD3100 alone can induce β-arrestin recruitment to CXCR7. Moreover, and in contrast to the antagonistic effect observed for CXCR4, AMD3100 increases ^125^I-CXCL12 binding to HEK293 cells expressing CXCR7 and CXCL12-facilitated recruitment of β-arrestin to CXCR7, recruitment that is also possible in the absence of CXCL12, albeit at relatively high concentrations (≥10 mM) [235]. To date and to the best of our knowledge, no molecular mechanism has been proposed to justify the agonistic property of AMD3100 on CXCR7.

Data about mode of action of AMD100 are limited in CRC. An in vitro study in the SW480 colon cell line demonstrated that the anti-tumor effect of AMD3100 was mediated through the reduction of VEGF and MMP-9 expression, but not MMP-2 [236]. Further evidence comes from a study on mammary stem cells that identified among the proteins showing CXCL12-induced phosphorylation, up to 22% are involved in signaling pathways related to cell adhesion and migration, actin and microtubule association in cytoskeletal remodeling. These mechanisms are known to support the involvement of CXCL12/CXCR4 in the metastatic process. By exposing cells to AMD3100, the phosphorylation of key proteins in these signaling pathways is blocked, such as the catalytic subunit of serine/threonine-protein phosphatase PP1-gamma (PPPC1) [237]. Conversely, in prostate cancer where CXCR4 strongly regulates the development of metastasis, treatment of prostate cells with dihydrotestosterone increased the expression of the androgen receptor, CXCR4, PI3K and AKT phosphorylation as well as EMT and downstream cell cycle control genes. Conversely, treatment with resveratrol and AMD3100 reversed all these changes associated with increased expression of apoptosis-related genes [238].

Taken together, these observations suggest that AMD3100 is an allosteric agonist to CXCR7. Therefore, while this antagonist has proved effective in controlling tumor progression in various cancers, these observations suggest caution in its use to understand the respective roles of CXCR4 and CXCR7 as mediators of the biological effects of CXCL12.

### 8.3. LY2510924

In addition to AMD3100, novel CXCR4 inhibitors have been identified, including the cyclic peptide LY2510924. From X-ray crystal structures of CXCR4 [239], LY2510924 is suggested to occupy a binding pocket and possess ligand–receptor interactions with CXCR4 residues such as Asp187, Arg188, Gln200, His113, and Tyr190 [240]. The antagonistic effect of this new molecule was confirmed in an SDF-1-induced GTP (guanine-triphosphate)-binding assay where LY2510924 completely inhibits SDF-1-mediated binding to GTPγS35 with a Kb of 0.38 nmol/L. Furthermore, LY2510924 was found to inhibit CXCL12-mediated chemotaxis by blocking SDF-1-stimulated phosphorylation of ERK and Akt in a concentration-dependent manner [240].

In vivo, its antagonistic effect has been proven by the dose-dependent decrease in tumor growth in colonic, pulmonary, renal or non-Hodgkin’s lymphoma xenografts and on the formation of mammary tumor metastases after intravenous injection of mammary tumor cells [240]. In the latter model, pre-treatment of mice with LY2510924 strongly decreases lung colonization and prevents the proliferation of implanted cells.

In a separate study, the inhibitory effects of LY2510924 were evaluated in orthotopic xenografts of three human colonic lines in the rectal mucosa. While treatment with LY2510924 strongly reduces tumor size, it does not affect the size of metastases and only when combined with 5-FU reduced metastasis [241]. A possible explanation for the lack of effect of LY2510924 on metastasis is the presence of a population of TICs, which is the source of many therapeutic resistances, or else this molecule is only fully effective when combined with other conventional therapies.

### 8.4. PepR

Peptide R (PepR) is a new CXCR4 antagonist peptide, effective mainly in combination with conventional chemotherapies such as 5-FU and oxaliplatin. In subcutaneous xenografts of HCT116 or HT-29 cells, mice treatment with PepR potentiates the inhibitory effect of chemotherapy on the proliferation and activation of EMT [242]. As a proposed mechanism, an analysis of TCGA dataset RNA-Seq indicates that adding the PepR compound to chemotherapy reverted the increased expression of the mesenchymal markers as well as PD-L1, all markers being induced by chemotherapy alone [242]. This suggests a role for CXCR4 in controlling EMT marker expression. In addition, treatment of colon cells with chemotherapy/radiochemotherapy induced a population of CD133+CXCR4+ cells, supposed to be stem-resistant cancer cells, while adding Pep R reduced this population. In a previous study, the same authors showed that this novel antagonist enhances the efficacy of anti-PD-1 therapy in a mouse model of colon cancer induced with MC38 cells [243]. The increased efficacy of anti-PD-1 therapy by PeR results from changes in the microenvironment by recruiting Granzyme B-positive cells and decreasing Tregs cells. Thus, PeR treatment makes the microenvironment more immunosensitive to anti-PD-1 therapy [243]. Other studies have shown that Pep R reduced the expression of CXCL12 and PD-L1, probably by inhibiting the immunosuppressive effect of the microenvironment and preventing the recruitment of stromal cells (CAFs, Tumor Associated Macrophages (TAM), Myeloid-Derived Suppressor Cells (MDSCs)) responsible for the exclusion of cytotoxic T lymphocytes approximately tumor cells [244,245].

### 8.5. MSX-122

Unlike other inhibitors that prevent the binding of CXCL12 to its receptor, this molecule MSX-122, when binding to CXCR4 could interfere with the “lock and key” mechanism between CXCR4 and CXCL12, and modulates functional signaling such as reductions in pErbB2, pAKT, pERK and increase in cAMP production, without displacing CXCL12 from the receptor [246].

The efficacy of this CXCR4 antagonist was evaluated in APC^Min/+^ mice exposed to azoxymethane (AOM) and treated with MSX-122 [247]. APC^Min/+^ mice are known to develop mainly small bowel tumors, while when exposed to AOM, they develop cancers in the colon [248]. As expected, AOM induced colonic tumors in these mice, whereas treatment with MSX-122 significantly reduced the incidence of colonic tumors and tumor volume through decreased cell proliferation as assessed by Ki-67 labeling. The authors propose that MSX-122, having been well tolerated in a phase Ib clinical trial, may serve as a chemopreventive agent in individuals at increased risk of developing CRC.

### 8.6. CCX754 and CCX771

In contrast to CXCR4, studies describing the use of CXCR7 antagonists in CRC are uncommon despite the development of several of its inhibitors by ChemoCentryx (CCX226, CCX733, CCX754, CCX771 and CCX773). These molecules have been described as ligands that do not induce phosphorylation of AKT or ERK. CCX754 and CCX771, two of these antagonists, were tested in mouse injected with human or mouse lung carcinoma cells [249] or in models of lung metastasis by injection of murine C26 and human HT-29 colon cancer cells [92]. Systemic treatment with CCX754 or CCX771 antagonist strongly reduced tumor expansion in the lungs of mice injected with these cells but not the expansion of metastases into the liver [92]. However, CCX771 has also been described as an agonist that recruits β-arrestin-2 to CXCR7 and blocks trans-endothelial migration of human cancer cells [250]. A theory of receptor desensitization has been proposed to explain the agonist/antagonistic effect of the molecule. CCX771 would not stimulate chemotactic activity but rather induce internalization of CXCR7 from the cell surface. This has been observed for a CCR5-targeting molecule in search for anti-HIV-1 agent [251] or described for a CCL7 agonist non-glycosaminoglycans (GAGs) binding and evaluated for its anti-inflammatory effect [252].

The lack of data on the efficacy of these antagonists can be explained by the following studies showing that those molecules initially designed to inhibit CXCR7 activation also act as agonists in different pathologies [253]. Likewise, some CXCR4 receptor antagonists are agonists for the CXCR7 receptor, such as the cyclic peptide TC14012 [254].

There may be several reasons why molecules presented as antagonists/agonists, exert inverse physiological activity. One possibility is that the mode of action of the molecules is more related to CXCL12-mediated effects than to CXCR7-mediated effects. For example, CXCR7 antagonists prevent CXCL12 internalization leading to increased extracellular CXCL12 concentrations. They may therefore generate pathophysiological effects such as those of CXCR7 agonists, as described in experimental autoimmune encephalomyelitis [255].

CCX771 alone induced a concentration-dependent association of CXCR7 with β-arrestin2 CCX771 was substantially more potent than its natural protein ligand CXCL12 in triggering β-arrestin2 association

### 8.7. Chalcones

In 2008, a screening of 3200 molecules from a medicinal library identified a new class of molecules that bind to the chemokine CXCL12 and act as neutral inhibitor of its biological activity in a way similar to neutralizing antibodies. The most potent compound which belongs to the chalcone family and named chalcone 4, has been shown to bind the chemokine CXCL12 with high affinity thus preventing the binding of the chemokine to both CXCR4 and CXCR7, and thus blocking the downstream pathways [256]. Later on, a study from our team demonstrated that chalcone 4 was able to reduce colorectal cell migration and when combined to irinotecan, further increased the inhibition [82]. However, this compound would need further characterization, yet no data has reported its capacity to block the dissemination process in vivo. CXCL12 is efficient in solubilizing chalcone molecules with a stoichiometry 3:1 for chalcone 4: CXCL12 and that chalcone 4 binds to one high affinity site and two low affinity sites in CXCL12 [256].

### 8.8. NOX-A12

Noxxon Pharma has developed a molecule called NOX-A12 or olaptesed pegol [257]. This molecule is an RNA aptamer (or spiegelmer), which acts by binding to CXCL12, preventing it from linking and activating its two receptors. It binds to CXCL12 with high affinity and specificity across various species such as humans, mice, and rats. NOX-A12 has been shown to bind directly to and inhibit CXCL12 but also detach the cell-surface bound CXCL12, leading to abrogation of the CXCL12 gradient [258]. In tissues, stromal cells secrete and present CXCL12 on the surface, via GAGs, and NOX-A12 can compete with GAGs to bind CXCL12, leading to the release of CXCL12 from the cell surface and thus neutralize the chemokine [258]. A study by Zboralski et al. showed in vitro that in tumor and stromal cell spheroids that mimic a solid tumor with a CXCL12-rich microenvironment, NOX-A12 promotes spheroid infiltration by T and NK cells in a dose-dependent manner. The combination of NOX-A12 and PD-1 checkpoint inhibitor acts synergistically to facilitate T cell infiltration into spheroids. These observations were validated in vivo in a mouse model of syngeneic CRC in which treatment with NOX-A12 improved the response to anti-PD-1 therapy to reduce tumor size [259]. A Phase I/II clinical trial is underway to study the effects of the NOX-A12 and anti-PD-1 combination in patients with advanced CRC or pancreatic carcinoma (NCT03168139).

**Table 2:** Chemical modulators of CXCR4 and CXCR7/ACKR3 activation.

Inhibitor/Antagonist	Formula	IC50	Target	References
AMD3100	1-[[4-(1,4,8,11 tetrazacyclotetradec-1-ylmethyl)phenyl]methyl]-1,4,8,11-tetrazacyclotetradecane	37.5 nM	CXCR4	[260]
LY2510924	N(1)Phe-D-Tyr-Lys(iPr)-D-Arg-2Nal-Gly-D-Glu(1)-Lys(iPr)-NH2	0.079 nM	CXCR4	[240]
PepR	(H-Arg-Ala-[Cys-Arg-Phe-Phe-Cys]-CO2H)	nd	CXCR4	[242,243]
MSX-122	N,N-9-(1,4-phenylenebis(methylene))dipyrimidin-2-amine	10 nM	CXCR4	[260]
CCX754	nd	5 nM	CXCR7	[249]
CCX771	nd	4.1 nM	CXCR7	[260]
Chalcone 4	((E)-1-(4′-chlorophenyl)-3-(4-hydroxy-3-metoxyphenyl) prop-2-en-1-one)	150 nM	CXCL12	[256]
NOX-A12	nd	5–200 nM	CXCL12	[257]

nd: not determined; IC50: 50% inhibitory concentration.

## 9. Clinical Trials

While many cases of CRC are diagnosed at an early stage and are treated with curative surgery, many patients develop synchronous or metachronous metastatic disease with a five-year survival rate of roughly 13% [261]. The routine treatment of metastatic CRC is based on the combination of different treatment schedules such as Folfiri/Folfox/Folfoxiri or Capiri/Capox, which resulted in a survival of about 18 months. However, more recently, the approval of targeted therapies with EGFR or VEGF antibodies has importantly improved the overall survival, approaching 30 months in clinical trials [262], but the relative unavailability of biomarkers in metastatic CRC has slowed the progress in tumor curacy. Because of the bad prognostic value of CXCR4 overexpression across different tumors, CXCR4-inhibition-based therapies have been therapeutically evaluated in hematologic and solid malignancies, either as monotherapy or in combination with chemotherapies or immunotherapies (for review, see [263,264,265]). Among the drugs tested in clinical trials, CXCR4 small molecule antagonists, fully humanized anti-CXCR4 antibodies and CXCR4 or CXCL12 peptide inhibitors represent the most advanced programs of CXCR4 inhibition in solid tumors. Galsky et al. published the first in-human phase I study in patients with advanced or metastatic CRC that explored the safety and tolerability of LY25110924 among other solid tumors [266]. To date, AMD3100 is the only approved CXCR4 inhibitor drug [231], while multiple antagonists are in different stages of development. Presently, the clinical trials are mainly ongoing phase I/II trials. They mainly concern the CXCR4 peptide inhibitor LY2510924 (NCT02737072), the anti-CXCR4 antibody LY2624587 (NCT01139788), the small molecule inhibitors Plerixafor (NCT20179970, NCT03277209), MSX-122 (NCT00591682, suspended), USL311 (NCT02765165); however, for a number of these trials, the cancer type is not always indicated, as it only specified that the targeted diseases are solid tumors.

The only available data from completed phase I/II trials evaluated the application of the NOX-A12 molecule (OPERA trial, NCT03168139), first as monotherapy, and then continued with pembrolizumab in patients with advanced stage pretreated metastatic colorectal or pancreatic cancer. The NOX-A12 was well tolerated and allowed for a disease control rate of 25%, and an overall survival close to 12 months could be achieved [267]. This effect was mediated by a transformation of the tumor immune microenvironment with the expression of a specific cytokine signature consisting of IL-2, IL-16 and IFN-γ as an indicator of activation in tumor tissue. Following this success, a phase II trial is currently underway to evaluate the effect of the combination of NOX-A12 and pembrolizumab in glioblastoma and pancreatic cancer.

Conversely, treating patients with CRC for seven days with continuous infusion of the CXCR4 inhibitor AMD3100/Plerixafor induces an integrated immune response with enhanced intratumoral immune B and T cell responses as observed in paired biopsies of metastatic lesions (NCT02179970) [268], an immune response that is predictive of a clinical response to T cell checkpoint inhibition. For other trials, no results are currently available, due to the required time to exploit the data.

Although several CXCR7 antagonists (CCX771, CCX662, CCX733, CCX754, and CCX777) have been investigated in preclinical models [253,269], to date, CXCR7 modulators have not been clinically investigated.

## 10. Resistance to Treatment

It is well established today that the increase in cancer mortality is partly due to the resistance of tumor cells to numerous anti-cancer treatments. Thus, understanding the mechanisms at the origin of this tumor resistance would lead to the development of new approaches to maximize the effectiveness of treatments. Two types of resistance are described in cancerology: innate resistance, which is a consequence of the high molecular heterogeneity of cells within a tumor, and resistance acquired during treatment [270]. In a tumor, inhibition of apoptotic signals promoting proliferation, DNA repair, genomic amplification, a defect in drug metabolism, or epigenetic modifications can generate acquired resistance [271,272]. Given the relevance of the CXCL12/CXCR4/CXCR7 axis in the development and progression of CRC, several studies have investigated its role in resistance to anti-cancer therapies.

In tumors, it is a common knowledge that a small population of cells known as Tumor Initiating Cells with stem cell characteristics are responsible for many tumor recurrences [273]. A subpopulation of tumor cells positive for TIC marker CD133 has been isolated from patient CRCs or colonic lines, and these cells are more tumorigenic than cells not sorted on marker expression CD133 [274]. Thus, the CD133+ cell population isolated and enriched for CXCR4 expression shows significant tumorigenicity with an increased in vitro cell proliferation, tumor size and angiogenesis in vivo [274,275]. By analyzing the secretion of soluble factors by the HK stromal ganglion cells, the authors found a significant expression of CXCL12, which by a paracrine action, promotes tumor vascular development and protects the cells from the therapeutic agents 5-FU and oxaliplatin [274,275].

Another study described co-expression of CXCR4 and Lgr5, a colonic stem cell marker receptor, in patients with stage IV CR [145]. In vitro, Caco-2 and HT-29 cells isolated by flow cytometry and strongly expressing CXCR4 and Lgr5 promote sphere formation and increase cell viability when treated with cytotoxic agents. Similarly in vivo, the concomitant expression of CXCR4/Lgr5 in cells implanted subcutaneously in mice confers a more important potential to develop a tumor mass [145].

In another study, combined treatment with endostar or endostatin (an angiogenesis inhibitor) and oxaliplatin synergistically decreased the proliferation, adhesion, and invasion of Matrigel [276]. This synergy is a consequence of decreased expression of CXCR4, as well as those of the hypoxic factors HIF-1α and HIF-2α [276]. The authors showed that only the accumulation of HIF-2α is responsible for this cell resistance to oxaliplatin, and the combination of endostar with oxaliplatin overcomes this resistance by making the cells more sensitive. These data suggest that CXCR4 could be used as a marker to identify tumor stem cell populations responsible for the resistance and recurrence seen in cancers.

MiRNAs, which are also involved in cancer pathology, are either tumor suppressors or oncomiRs, largely involved in proliferation, invasion, and resistance to treatment. Some miRNAs are targets of the CXCL12/CXCR4/CXCR7 axis, and one study investigated the role of miR-125b in 5-FU resistance of cells expressing CXCR4 [182]. Expression of miR-125b, increased by the treatment of HCT116 cells with CXCL12, accelerates invasive ability and promotes EMT, which in turn increases CXCR4 expression, forming a reciprocal positive feedback loop between CXCR4 and miR-125b. Upregulation of miR-125b also activates Wnt/β-catenin signaling and the APC gene and contributes to 5-FU resistance by enhancing cellular autophagy [182].

Contrary to these studies, Heckmann et al. described that overexpression of CXCR4 in the SW480 colonic line and strong endogenous expression in HT-29 cells is associated with a higher sensitivity to treatments such as 5-FU, oxaliplatin or irinotecan. This chemosensitivity, assessed by a decrease in cell survival, cytotoxicity, and apoptosis, is further increased when one of these molecules is combined with plerixafor [277]. In this case, contrary to CXCR4, it would rather be the overexpression of CXCR7 that results in the resistance [278].

## 11. Conclusions

**Figure 2:**
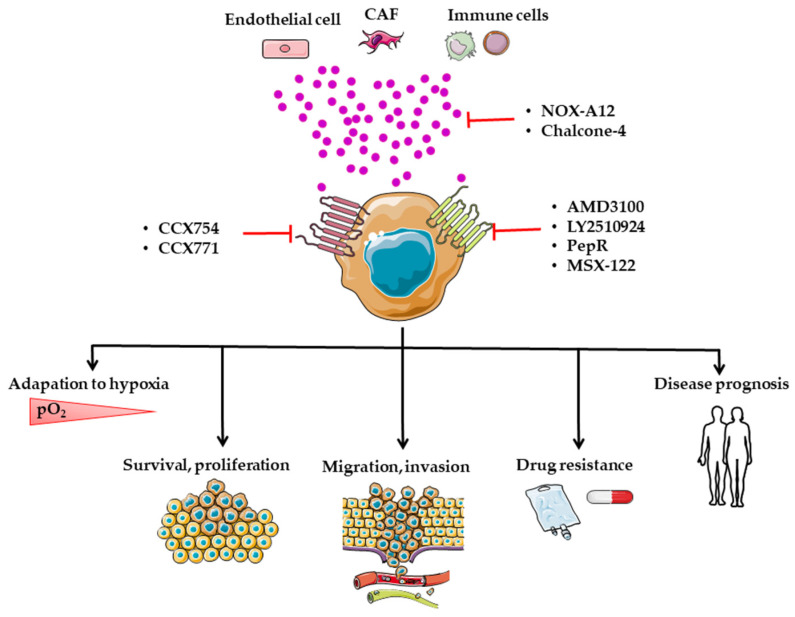
Involvement of the CXCL12/CXCR4/CXCR7 axis in regulating primary tumor growth and metastasis and its pharmaceutical targeting. The expression levels of either partner of this axis have a prognostic value and participate in tumor progression through the activation of multiple signaling pathways involved in cell survival, proliferation, invasion, and migration/dissemination. Each step of the process can be activated/facilitated by local hypoxia within the primary tumor. At sites of metastasis, CXCL12-producing cells (endothelial cells, CAFs, immune cells) allow for the implantation of receptor-expressing tumor cells. In colorectal cancer, several therapeutic molecules targeting receptors or chemokines are undergoing clinical trials to improve patient management and/or overcome tumor resistance. Pink dots indicate CXCL12 molecules.

In CRC, activation of the CXCL12/CXCR4/CXCR7 axis leads to progression and development of metastases with an unfavorable disease outcome and poor patient survival. Disruption of the CXCL12-CXCR4/CXCR7 axis remains an interesting target for pharmacological treatment (Figure 2). CXCR4 and CXCR7 antagonists are being tested in several preclinical and clinical trials for the treatment of CRC, and other gastrointestinal cancers, but with limited success and the development of combined antagonists, targeting both receptors are still lacking. Therefore, tumor immunotherapy entered a phase of rapid development in cancer treatments, but there are too many patients resistant to this therapy. Furthermore, the use of inhibitors targeting the oncogenic CXCL12 axis in combination with current immunotherapies should be considered and may provide hope for improving cancer treatments.

## Abbreviations

ACKR3	atypical chemokine receptor 3
Akt	protein kinase B
cAMP	cyclic adenosine monophosphate
AOM	azoxymethane
APC	adenomatous polyposis coli
ARE	AU-rich element
ChIP	chromatin immunoprecipitation
CIN	chromosome instability
CIMP	CpG island methylator phenotype
CpG	cytosine-phosphate-guanine
CRC	colorectal cancer
CTLA-4	cytotoxic T lymphocyte Antigen 4
CXCL-10/12	C-X-C motif ligand 10/12
CXCR-3/4/7	C-X-C motif receptor 3/4/7
CAF	cancer-associated fibroblasts
DSS	dextran sodium sulfate
ECM	extracellular matrix
EGFR	epidermal growth factor receptor
EMT	epithelial–mesenchymal transition
ERK	extracellular signal-regulated kinases
FAP	familial adenomatous polyposis
FGF	fibroblast growth factor
5-FU	5-fluorouracil
GAG	glycosaminoglycans
GEO	Gene Expression Omnibus
GRK2	G-protein-coupled receptor kinase 2
GTP	guanine–triphosphate
HDAC	histone deacetylases
HIC1	hypermethylated in cancer 1
HIF	hypoxia-inducible factor
HiRE	HIC1-responsive elements
HIV	human immunodeficiency virus
IBD	chronic inflammatory bowel disease
ICAM	intercellular adhesion molecule
ICI	immune checkpoint inhibitor
IGF	insulin-like growth factor
IL-2/6	interleukin-2/6
ITG	integrin
Jak	Janus kinase
JNK	c-Jun N-terminal kinase
Lgr5	leucine-rich repeat-containing G-protein coupled receptor 5
MAPK	mitogen-activated protein kinases
MDSCs	myeloid-derived suppressor cells
MMP	matrix metalloproteinase
MMR	mismatch Repair
MSC	mesenchymal stromal cells
MSS	microsatellite stability
MSI	microsatellite instability
mTOR	mammalian target of rapamycin
NF-κB	nuclear factor-kappa B
PCAF	P300/CBP-associated factor
PDGF	platelet-derived growth factor
PD-1	programmed cell death protein 1
PD-L1	programmed cell death protein ligand 1
PI3K	phosphatidylinositol 3-kinase
PLC	Phospholipase C
PPPC1	serine/threonine-protein phosphatase PP1-gamma catalytic
SDF-1	stromal-cell-derived factor 1
α-SMA	alpha-smooth muscle actin
STAT	signal transducer and activator of transcription
TAM	tumor associated macrophages
TCGA	the cancer genome atlas program
TGF	tumor growth factor
TIC	tumor-initiating cell
TIMP	tissue inhibitor of metalloproteinases
TNF	tumor necrosis factor
TNM	tumor, node, metastasis
TTP	tristetraprolin
UTR	untranslated transcribed region
VEGF	vascular endothelial growth factor
